# Cardiac Surgery Costs According to the Preoperative Risk in the Brazilian Public Health System

**DOI:** 10.5935/abc.20150068

**Published:** 2015-08

**Authors:** David Provenzale Titinger, Luiz Augusto Ferreira Lisboa, Bruna La Regina Matrangolo, Luis Roberto Palma Dallan, Luis Alberto Oliveira Dallan, Evelinda Marramon Trindade, Ivone Eckl, Roberto Kalil Filho, Omar Asdrúbal Vilca Mejía, Fabio Biscegli Jatene

**Affiliations:** Instituto do Coração do Hospital das Clínicas da Faculdade de Medicina da Universidade de São Paulo, São Paulo, SP – Brazil

**Keywords:** Cardiac Surgical Procedures / economics, Hospital Costs, Unified Health System, Risk Groups, Preoperative Care, Hospital Mortality, Morbidity

## Abstract

**Background:**

Heart surgery has developed with increasing patient complexity.

**Objective:**

To assess the use of resources and real costs stratified by risk factors of patients submitted to surgical cardiac procedures and to compare them with the values reimbursed by the Brazilian Unified Health System (SUS).

**Method:**

All cardiac surgery procedures performed between January and July 2013 in a tertiary referral center were analyzed. Demographic and clinical data allowed the calculation of the value reimbursed by the Brazilian SUS. Patients were stratified as low, intermediate and high-risk categories according to the EuroSCORE. Clinical outcomes, use of resources and costs (real costs versus SUS) were compared between established risk groups.

**Results:**

Postoperative mortality rates of low, intermediate and high-risk EuroSCORE risk strata showed a significant linear positive correlation (EuroSCORE: 3.8%, 10%, and 25%; p < 0.0001), as well as occurrence of any postoperative complication EuroSCORE: 13.7%, 20.7%, and 30.8%, respectively; p = 0.006). Accordingly, length-of-stay increased from 20.9 days to 24.8 and 29.2 days (p < 0.001). The real cost was parallel to increased resource use according to EuroSCORE risk strata (R$ 27.116,00 ± R$ 13.928,00 versus R$ 34.854,00 ± R$ 27.814,00 versus R$ 43.234,00 ± R$ 26.009,00, respectively; p < 0.001). SUS reimbursement also increased (R$ 14.306,00 ± R$ 4.571,00 versus R$ 16.217,00 ± R$ 7.298,00 versus R$ 19.548,00 ± R$935,00; p < 0.001). However, as the EuroSCORE increased, there was significant difference (p < 0.0001) between the real cost increasing slope and the SUS reimbursement elevation per EuroSCORE risk strata.

**Conclusion:**

Higher EuroSCORE was related to higher postoperative mortality, complications, length of stay, and costs. Although SUS reimbursement increased according to risk, it was not proportional to real costs.

## Introduction

During the last four decades, cardiac surgery has developed with the increase in complex procedures in progressively critically-ill patients^1^. Evidence shows that this scenario proportionally increases with morbimortality and hospital costs^2,3^.

In Brazil, most of the highly complex procedures are performed with funding from the Unified Health System (SUS). This system is responsible for 80% of CABG surgeries performed in the country^4^. The reimbursement for hospitals that belong to SUS uses SUS own price list for the Hospitalization Authorization (AIH). The payment of this value is little yielding in its composition and may not reflect correct fund allocation that correspond to the Actual Cost (AC) of the procedure^5^. Thus, the AIH paid by SUS for the procedure may have no direct association with patient severity.

An unequal association between the AC of these procedures and SUS reimbursement may discourage hospital care provided to high-risk surgical patients, which are the cases that would benefit the most from these procedures^6^.

On the other hand, international guidelines advise about the use of risk scores to identify patients at higher risk of morbimortality^7^. In Brazil, the EuroSCORE is the most used model and the only one that has been validated in significant samples^8^.

The objective of this study was to evaluate the use of resources by risk group, comparing the AC of cardiovascular procedures with SUS reimbursement in a hospital.

## Methods

### Sample

A prospective observational study carried out at the Cardiovascular Surgery Division and SUS Billing Unit of Instituto do Coração do Hospital das Clínicas da Faculdade de Medicina da Universidade de São Paulo (InCor-HC/FMUSP) (Heart Institute of the Faculty of Medicine, University of São Paulo (InCor-HC / USP)).

Data from consecutive patients were obtained from the institution’s database (SI3). All demographic data that could identify patients were removed. Clinical data and use of resources were exported to an Excel spreadsheet for analysis. By cross-checking patients’ data with the registry of the participating units, it was verified that there were no errors and no patients loss due to lack of data.

### Inclusion and exclusion criteria

The inclusion criteria were: adult patients consecutively operated between January and July 2013, in the elective, urgent or emergency mode, at the Cardiovascular Surgery Division of InCor-HC/FMUSP.

Patients not hospitalized by SUS were excluded from the study.

### Data collection, definition and organization

Data were collected prospectively in the electronic medical file system of InCor-HC/FMUSP (SI3). After exporting data to a single worksheet in Excel and removal of demographic data that could identify patients, this worksheet was adapted to take into account all the variables described in the first EuroSCORE model (additive version)^9^. All definitions assigned to variables by EuroSCORE were accomplished, together with their values, according to their relevance to the death event.

Therefore, after calculating the value of the variables in each patient, the patients were classified according to the risk groups established by the model. In addition to the clinical and laboratory variables included in the EuroSCORE, the economic variables were considered. The total value of AC included fixed and variable costs per patient. The AC was calculated by analysis of variable costs accounted by the micro-costing methodology^10^ and by the full costing method for the fixed costs. The mean unit cost of each material item and medications was estimated from the purchases of these items during this period, being considered, in each category, the individual units costs. The mean unit cost of each diagnostic service, daily hospital stay costs or therapy was estimated by total inclusion of fixed costs (pro-rata of general consumption fixed costs – water, electricity and telephone, auxiliary services – maintenance contracts, cleaning services, air conditioning, etc., and administrative services) from the cost centers, divided by unit of produced outcome. Thus, we considered the quantitative variables ICU length of stay (days), hospitalization length of stay (days) and time of orotracheal intubation (hours). Similarly, it was considered the total value of SUS reimbursement, adding hospital service, professional service, ICU and compatible materials.

The primary outcomes were in-hospital mortality and morbidity (cerebrovascular accident, Renal Replacement Therapy - RRT, pneumonia, atrial fibrillation, mediastinitis/osteomyelitis and reoperation for bleeding). The definitions of the study variables outside the EuroSCORE were taken from the glossary of the American Heart Association^11^. All patients were followed until hospital discharge.

### Statistical analysis

Continuous variables were expressed as mean ± standard deviation or median, and categorical variables as percentages. Logistic regression analysis for the hospital morbidity and mortality outcome was performed by using the value provided by EuroSCORE for each patient. Patients were subdivided by the EuroSCORE as low (1-4), intermediate (5-7), and high (≥ 8) risk.

The three categories were analyzed to highlight the differences related to the morbimortality, resource use, AC and SUS reimbursement. Variable distribution was tested for normality using the Kolmogorov-Smirnov test. Variables with normal distribution were compared between the risk categories using analysis of variance. Paired comparisons were corrected using the Bonferroni-Dunn test.

Student’s *t* test was used for parametric distributions, and the Mann-Whitney and Kruskal-Wallis tests were used for non-parametric distributions. Categorical variables were compared using Pearson’s chi-square test. The null hypothesis was rejected when p < 5% (p < 0.05). This study made a comparison, in the “real world”, between the mean costs of the risk categories, reducing the possibility of bias in patient selection^12^. The analysis was performed using the Statistical Package for the Social Sciences (SPSS) software, version 20.0.0 (Chicago, IL).

### Ethics and Consent Form

This study was approved by the Ethics Committee for Analysis of Research Projects (CAPPesq) HC/FMUSP, under number 1575, being exempt from the need to use the Free and Informed Consent form, due to the use of analysis of non-identified data only.

## Results

### Sample

The characteristics of patients in the different risk groups are shown in Table 1. The low-risk group consisted of 131 (34%) patients, the intermediate risk group, of 150 (39%) and the high-risk group of 104 (27 %) patients. There were significant differences in EuroSCORE means according to the risk group: 2.91 ± 1.03, 5.89 ± 0.84 and 10.32 ± 2.6 in the lower, intermediate and high-risk categories, respectively. The mean age was 61 ± 12.29 years.

**Table 1:** Patient characteristics

Characteristics	Sample (n = 385)	Low Risk (n = 131)	Intermediate Risk (n = 150)	High Risk (n = 104)	p
Age	61 ± 12.3	56.1 ± 10.3	61.2 ± 12.5	65.3 ± 12.5	< 0.001*
Female gender	160 (41.6)	48 (36.6)	69 (46)	43 (41.3)	0.28
EuroSCORE	6.1 ± 3.3	2.9 ± 1	5.9 ± 0.8	10.3 ± 2.6	< 0.001*
Creatinine > 2mg/dL	39 (10.1)	2 (1.5)	11 (7.3)	26 (25)	< 0.001‡
Ejection fraction < 30%	32 (8.3)	7 (5.3)	16 (10.7)	9 (8.7)	0.27
Recent Infarction	42 (10.9)	7 (5.3)	18 (12)	17 (16.3)	0.012†
Reoperation	68 (17.7)	5 (3.8)	25 (16.7)	38 (36.5)	< 0.001*
CABG	188 (48.8)	78 (59.5)	78 (52)	32 (30.8)	< 0.001‡
HVS	173 (44.9)	50 (38.2)	63 (42)	60 (57.7)	0.002‡
CABG + HVS	21 (5.5)	3 (2.3)	7 (4.7)	11 (10.6)	0.007‡
Others (Not CABG + HVS)	3 (0.8)	0	1 (0.7)	2 (1.9)	0.28
Urgency / Emergency	17 (4.4)	2 (1.5)	6 (4)	9 (8.7)	0.014‡
Events	38 (9.9)	0	4 (2.7)	34 (32.7)	< 0.001‡

The item “events” includes at least one of the following situations prior to surgery: intra-aortic balloon, cardiogenic shock, ventricular tachycardia or fibrillation, orotracheal intubation, acute renal failure, use of inotropic drugs and cardiac massage.

* Significant difference between all risk groups;

† significant difference between the low/intermediate risk groups and the high-risk group;

‡ significant difference between the low-risk group and the intermediate/high risk group. CABG: coronary-artery bypass grafting; HVS: heart valve surgery.

### Clinical outcomes and resource use

**Figure 1:**
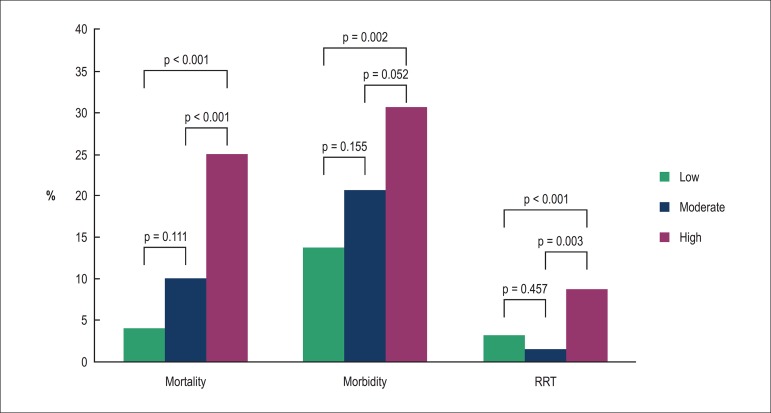
In-hospital outcomes of morbidity, mortality and renal replacement therapy (RRT) by risk groups, according to the EuroSCORE

The clinical outcomes and resource use are shown in Table 2. The EuroSCORE was associated with death (p < 0.0001) and showed good calibration (p = 0.9744) in the Hosmer-Lemeshow test. Nevertheless, this model was associated with morbimortality (p < 0.0001) and also showed good calibration (p = 0.2221) in the Hosmer-Lemeshow test. Mortality, morbidity and morbimortality of 11.26, 21.41 and 27.15% were observed, respectively. There was 3.82% of mortality in low risk, 10% in intermediate risk and 25% in high risk (Figure 1). The low-risk group had the lowest percentage of deaths, which increased with the risk increase (p < 0.0001). There was 13.74% of morbidity in low risk, 20.67% in intermediate risk and 30.77% in the high risk. The low-risk group had a lower percentage of complications, which increased with the risk increase (p = 0.0063). There was 3.1% of RST in the low-risk, 1.3% in intermediate-risk and 8.7% in the high-risk group. The low-risk and intermediate-risk groups had the lowest percentage of RST, and the high-risk group had the highest percentage (p = 0.003). While one can observe that, regarding the length of stay, the high-risk group showed no significant difference compared to the intermediate risk, the low-risk group had a significantly lower value than the groups at high and intermediate risk. In the analysis of ICU length of stay, we observed that the low-risk group had significantly lower value than the groups with intermediate and high risk, and the group with intermediate risk had a significantly lower value than the high-risk group.

**Table 2:** Clinical outcomes and resource utilization

Variable	Sample (n = 385)	Low risk (n = 131)	Intermediate risk (n = 150)	High risk (n = 104)	p
Mortality	56 (14.5)	5 (3.8)	15 (10)	26 (25)	< 0.001*
Morbidity	81 (21)	18 (13.7)	31 (20.7)	32 (30.8)	0.004*
CVA	1 (0.3)	1 (0.8)	0	0	0.61
Atrial fibrillation	30 (7.8)	6 (4.6)	15 (10)	9 (8.7)	0.22
RRT	15 (3.9)	4 (3.1)	2 (1.3)	9 (8.7)	0.003*
Pneumonia	12 (3.1)	4 (3.1)	3 (2)	5 (4.8)	0.46
Reoperation x Bleeding	17 (4.4)	5(3.8)	4 (2.7)	8 (7.7)	0.15
OTI>24h	22 (5.7)	3 (2.3)	9 (6)	10 (9.6)	0.055
Time of ICU	8.3 ± 10.1 days	5.6 ± 5.9 days	8.1 ± 10.4 days	11.9 ± 12.6 days	< 0.001‡
Hospital length of stay	25 ± 17days	21 ± 13.2days	25 ± 13.25 days	29 ± 16.3days	< 0.001†

* Significant difference between low / intermediate risk and high risk groups.

† Significant difference between the low risk and intermediate / high risk groups.

‡ Significant difference between all risk groups. CVA: cerebrovascular accident; RRT: renal replacement therapy; OTI: orotracheal intubation; ICU: intensive care unit.

### SUS reimbursement and actual cost

**Figure 2:**
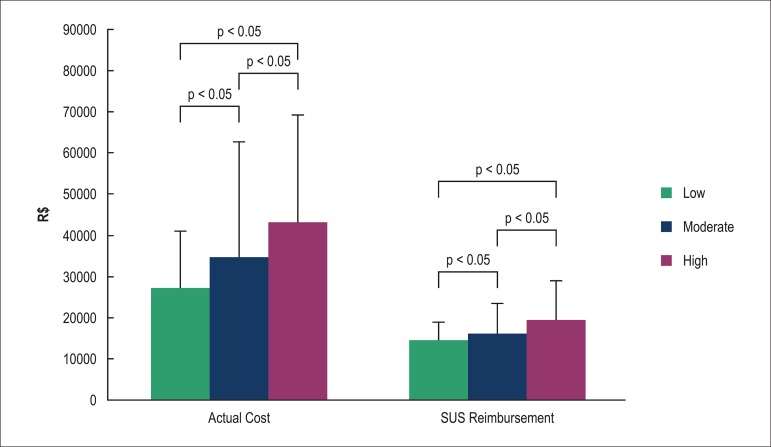
Total value of actual costs and the Unified Health System (SUS) reimbursement for risk groups, according to EuroSCORE.

Risk groups differed in relation to the total value of the SUS reimbursement (low risk: R$ 14.306,00 ± R$ 4.571,00; intermediate risk: R$ 16.115,00 ± R$ 7.381,00, and high risk: R$ 19.548,00 ± R$ 9.355,00, p < 0.001), being higher in higher risk categories. Still, regarding the AC, the low risk group (R$ 27.116,00 ± R$ 13.928,00) showed a significantly lower value than the other groups, and the intermediate risk group had a significantly lower value than the high group risk (R$ 34,854.00 ± R$ 27,814.00 & R$ 43.234,00 ± R$ 26.009,00 ± R) (Figure 2).

**Figure 3:**
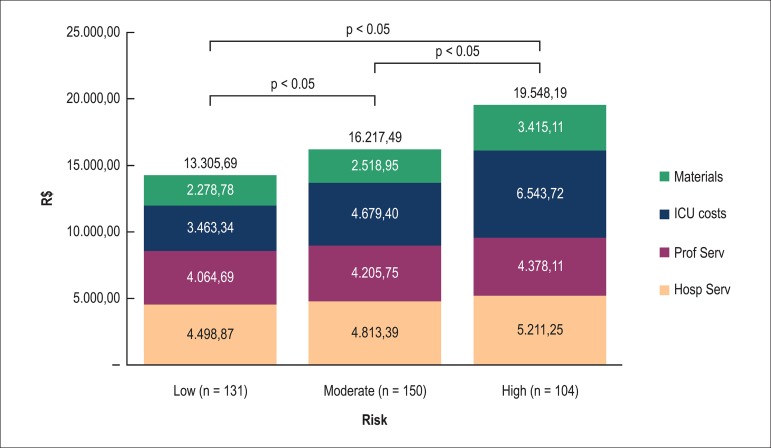
Detailed values of the of the Unified Health System (SUS) reimbursement for cardiovascular procedures by risk groups, according to EuroSCORE. Materials: reimbursement for cost of materials (excluding drugs); ICU costs: reimbursement for the intensive care unit services; Prof Serv: reimbursement for professional services; Hosp Serv: reimbursement for hospital services

However, when we analyze the risk groups for specific values of SUS reimbursement, we found some discrepancies not demonstrated in the total sample (Figure 3). In reimbursement for hospital services, even if the high-risk group had significantly higher value than the low and intermediate-risk groups, the low-risk group showed no significant difference in relation to the intermediate risk group. Similarly, in relation to reimbursement for professional services, the low-risk group showed no significant difference in relation to the intermediate-risk group, although there was a lower significant difference in relation to the high-risk group.

In this item, the intermediate and high-risk groups were not significantly different. Similarly, on the reimbursement for the cost of materials, even if the high-risk group had significantly higher value than the low and intermediate-risk groups, the low risk group showed no significant difference in relation to the intermediate risk. Only in the reimbursement assessment for the ICU costs, the low-risk group had significantly lower value than the groups with intermediate and high risk, and the group with intermediate risk had a significantly lower value than the high-risk group.

**Figure 4:**
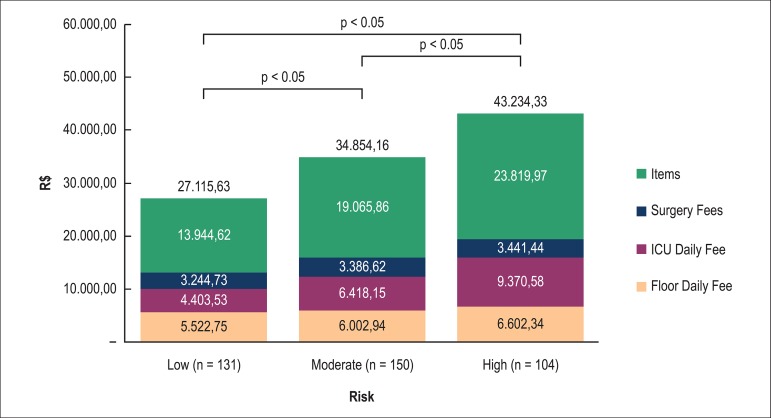
Detailed values of the actual costs for cardiovascular procedures by risk groups, according to the EuroSCORE. Items: includes the actual value of the materials and drugs; ICU: intensive care unit

However, when we discriminately analyze the items established for the AC calculation (Figure 4), we can observe a significant difference as the risk increases by EuroSCORE.

To confirm this, a logistic regression model was created for the SUS reimbursement value *versus* EuroSCORE (p < 0.0001):

11371 + 839.14*EuroSCORE

It was also a model for the AC value *versus* EuroSCORE (p < 0.0001):

18831 + 2577.69*EuroSCORE

Thus, with the estimates obtained from EuroSCORE (Table 3), the greater the patient risk, the greater the difference between the AC and the SUS reimbursement value.

**Table 3:** Estimates obtained from the regression models for reimbursement by the Unified Health System (SUS) and the actual cost (AC), according to the EuroSCORE value.

EuroSCORE	SUS (R$)	AC (R$)	Difference (R$)
0	11,371.00	18,831.00	-7,460.00
2	13,049.28	23,986.38	-10,937.10
4	14,727.56	29,141.76	-14,414.20
6	16,405.84	34,297.14	-17,891.30
8	18,084.12	39,452.52	-21,368.40
10	19,762.40	44,607.90	-24,845.50
12	21,440.68	49,763.28	-28,322.60
14	23,118.96	54,918.66	-31,799.70
16	24,797.24	60,074.04	-35,276.80
18	26,475.52	65,229.42	-38,753.90

(R$) Values in Brazilian reais.

## Discussion

Being a reference only in simple procedures should not give credit to an institution that does not make any effort to treat critically-ill patients that need complex surgeries. With an aging population and increasing life expectancy^13^, a larger population of frail patients is referred for cardiovascular procedures and improved quality of life. Evidence shows that critical patients are those that benefit the most from cardiovascular procedures, even if they have higher cost and morbimortality risk^14^.

This would explain why surgeons and hospitals that accept to operate more severe patients can have higher costs and greater morbimortality^15,16^. The use of risk scores allows the correction of the results according to patient severity for a more stringent cost-effectiveness analysis^17^. In Brazil, the most widely used risk model in cardiovascular surgery for outcome adjustment is the EuroSCORE^18-22^. Our study confirmed the direct association of the EuroSCORE with increased mortality and morbimortality.

SUS performs the majority of cardiovascular surgeries in Brazil, treating primarily patients with more unfavorable socioeconomic conditions. At InCor-HC/FMUSP, the number of cardiovascular surgeries by SUS corresponds to approximately 80% of the total. It is important to mention that the government allocates to the public health in Brazil a total of US$ 157.00 per inhabitant/year (I/Y). This is in sharp contrast with public health spending in Germany (US$ 3.521,00 I/Y), Canada (US$ 2.823,00 I/Y), United States (US$ 2.725,00 I/Y), Portugal (US$ 1,850,00 I/Y), Chile (US$ 720,00 I/Y), Argentina (US$ 380,00 I/Y) and Costa Rica (US$ 378,00 I/Y)^6^. We know that the value of public spending in the US is an emblematic example of a system segmented for the poor (*Medicaid*), elderly (*Medicare*) and war veterans (about 66 million of inhabitants), while Brazil is the source of funding for approximately 160 million of inhabitants^23^.

A publication on patients undergoing aortic valve replacement in the United States showed a direct correlation between the risk increase of patients and increased morbimortality and costs^14^. In Brazil, a study published by Instituto Dante Pazzanese de Cardiologia^5^ (Dante Pazzanese Institute of Cardiology) showed that the cost of coronary artery bypass surgery (primary, isolated and elective) is lower than the reimbursement supplied by SUS, showing that the mean cost of surgery was R$ 6.990,00 and the amount paid was R$ 5.551.41. These values are different from those found in our analysis, upon which the variety of procedures performed, including emergency care, the progressive worsening of the patients over time, and the current adjustment of costs and SUS reimbursement may have influenced.

This cost discrepancy has made university and philanthropic hospitals, and even private hospitals with social security-funded care suspend medical care due to accumulated debt. All this can worsen considering the global trend of increased high-risk patients referred to undergo cardiovascular procedures.

**Figure 5:**
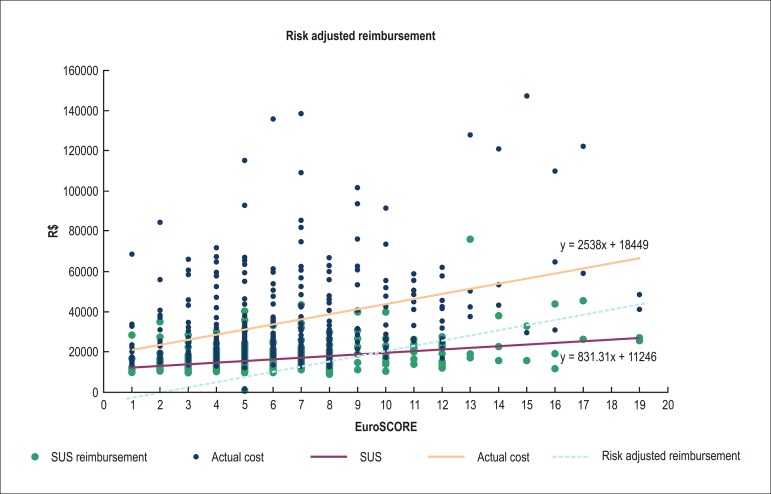
Unified Health System (SUS) reimbursement increase, actual cost (AC) and risk adjusted reimbursement, according to the EuroSCORE value

In this study, it was shown that AC increases progressively when the preoperative risk of the patient increases. Although the SUS reimbursement also increases with the patient’s risk, it is disproportionate to the AC, and this increases as the patient’s risk increases. This scenario could influence the selection of patients operated in SUS-funded hospitals. Unquestionably, the ideal would be that SUS-funded hospitals be reimbursed by an amount equivalent to the AC. However, the minimum to be done is a reimbursement proportional to the AC. In the current context and for the same budget, that would be to pay less for low-risk surgeries and more for higher-risk surgeries, according to what we call risk adjusted reimbursement (Figure 5). Therefore, for each EuroSCORE unit increase, there will be a fairer amount to be reimbursed by SUS.

### Study limitations

There are several limitations in this study. First, no follow-up was performed for long-term analysis, although a recent study showed that, in a follow-up of five years after aortic valve replacement, there was a higher cost for high-risk patients^24^. Second, a multicenter analysis could have found differences related to specific patterns of SUS reimbursement between hospital categories. Third, the sample size may have influenced some analyses, especially among the categories of intermediate and high risk. Fourth, some risk factors, such as frailty, were excluded from the study. However, this could increase differences in the high-risk patient group^25^.

In short, high-risk patients referred for cardiovascular surgery, in addition to the fact that they have higher cost, also show higher risk of morbimortality. Analyses in larger samples are needed to justify the cost-effectiveness of the procedures, to support SUS sustainability and funding, and improve the quality of outcomes and safety for patients.

## Conclusions

Although the SUS reimbursement increases with the increase in patient risk, it is disproportionate to the real cost. Future directions in SUS reimbursement should be adopted so that care of an increasing number of high-risk surgical patients is not discouraged.
